# Status of Medications Prescribed for Psychiatric Disorders in Korean Pediatric and Adolescent Patients

**DOI:** 10.3390/children9010068

**Published:** 2022-01-05

**Authors:** In-Woo Jang, Ji-Eun Chang, Jongyoon Kim, Kiyon Rhew

**Affiliations:** College of Pharmacy, Dongduk Women’s University, Seoul 02748, Korea; cattuhr@gmail.com (I.-W.J.); jieun0515@dongduk.ac.kr (J.-E.C.); jyoonkim@dongduk.ac.kr (J.K.)

**Keywords:** pediatrics, psychiatric drugs, off-label, unlicensed, real-world evidence, real-world data

## Abstract

While mental health services for children are increasing, few psychiatric drugs have been approved for such use. We analyzed claim data from 19,557 South Korean pediatric and adolescent patients (<20 years) who were diagnosed with schizophrenia, bipolar disorder, major depressive disorder, anxiety disorder, attention deficit-hyperactivity disorder (ADHD), or a tic disorder. Among these diseases, depressive episodes were the most common, followed by an anxiety disorder, ADHD, bipolar disorder, tic disorder, and schizophrenia. For each disease, prescriptions were categorized as full-label (approved indication with pediatric dosing in the package insert (PI)), partial-label (approved indication without pediatric dosing in the PI), and contraindication (contraindicated for the specific pediatric age in the PI). For schizophrenia, major depressive disorder, and anxiety disorder, more than 50% of the patients were prescribed partial-labeled medications. Additionally, more than 5% of patients with major depressive disorder were prescribed medications that were contraindicated for their age group. Our findings reveal that children with full-labeled psychiatric conditions are commonly administered drugs that are not explicitly approved for either their disease state or age, including off-label and unlicensed drugs. To use pharmaceuticals more safely, expanding drug indications using real-world data are needed.

## 1. Introduction

The prevalence of mental illness in children and adolescents worldwide and the use of medical services to treat such illnesses are increasing [1,2,3]. In the United States, attention deficit-hyperactivity disorder (ADHD) is diagnosed in about 10% of children under the age of 18 [4], and 7.4%, 7.1%, and 3.2% of children and adolescents are diagnosed with behavioral disorders, anxiety disorders, and depressive disorders, respectively [5]. Due to the increasing prevalence of mental health disorders in children and adolescents, research related to these disorders is also increasing.

Few drugs have received regulatory approval to treat these disorders in children and adolescents. According to a study published in 2017, between 36.3% and 97% of drugs prescribed for children and adolescents to treat mental health disorders are used off-label, and 18.6% to 40.2% are unlicensed [6]. In a study conducted in Sweden, psychoactive agents were most prescribed and used off-label for children and adolescents to treat mental health disorders [7]. Further, in Denmark, 27.6% of prescription drugs used for psychiatric disorders in children and adolescents are used off-label [8], and their use in Asia is no different. In a study in China, about 75% of the drugs prescribed to treat mental health disorders in children and adolescents are not approved by the China Food and Drug Administration, a regulatory foundation in China, and 15% are reportedly used off-label [9]. Among them, the most frequently prescribed drug was olanzapine, accounting for about 25% of all prescriptions for pediatric mental health disorders [5].

These results may simply reflect that many unapproved drugs are used to treat mental illnesses, but they can also indicate a lack of drugs with clear and sufficient indications for the treatment of specific mental illnesses in children and adolescents. Therefore, it is meaningful to analyze the current status of medicines prescribed for mental health disorders that are frequently diagnosed in children and adolescents in Korea as well as to examine the current status of drug prescriptions according to their approved indications. These findings can then be used as a future reference for effective and safe drug use and as a basis for preparing real-world evidence (RWE) based on real-world data (RWD).

Thus, this study analyzed the current status of medicines used to treat children and adolescents with psychiatric disorders, rather than the use of nonpharmacological treatment approaches such as cognitive-behavioral therapy, as well as the application of such medicines to standards that may be unclear, such as off-label or non-approved drugs.

## 2. Materials and Methods

### 2.1. Analytical and Patient Data

In this study, a pediatric patient data set (HIRA-PPS-2018) provided by the Health Insurance Review and Assessment Service (HIRA) was used. These data were constructed by stratifying 10% (approximately one million) of the pediatric and adolescent patients (under 20 years of age) by age and gender, targeting patients who were examined at a medical institution more than once in 2018. The inclusion criteria included at least a single diagnosis of schizophrenia (F20-23, F25), bipolar disorder (F31), depressive disorder (F32, F33), anxiety disorder (F41), ADHD (F90), or tic disorder (F95), as defined by the disease code. This study was conducted under the review (DDWU210802) of the Institutional Ethics Review Committee of Dongduk Women’s University.

### 2.2. Classification Criteria for Drugs by Indication

We prepared a list of drugs that could be prescribed for children and adolescents, as suggested by the domestic and international guidelines for mental health disorders; based on this list, drugs approved by the Ministry of Food and Drug Safety in Korea were included as the target drugs for this study [10,11,12,13,14,15,16,17,18]. Since the number of drugs approved to treat mental health disorders in pediatric and adolescent patients was limited, we classified drugs by indication, based on the information presented in the approval list: (1) Full-label—drugs approved indication with pediatric and adolescent dosing in the package insert (PI). In these cases, the indications (efficacy/effect) and usage/dose of the drug according to the age of the patient were presented in the approval information; (2) Partial-label—approved indication without pediatric and adolescent dosing in the PI; and (3) Contraindication—contraindicated for the specific pediatric and adolescent age in the PI. The lists of drugs are in Table 1.

The HIRA pediatric dataset provided patient’s age in two category variables: (1) pediatric age group (0–2 years, 3–5 years, 6–9 years, 10–12 years, or 13–19 years) and (2) age groups with five-year intervals. Therefore, if age were suggested when classifying drugs based on indication, the drugs were classified using these categories. Contraindicated drugs, according to the limitations of the age information provided by the HIRA dataset, are as follows: (1) Perphenazine is contraindicated for use in children under 14 years of age; however, its use was recorded in children under 12 years of age. (2) The use of diazepam is contraindicated in infants under 6 months of age. In this study, its use in children under 2 years of age was considered contraindicated.

### 2.3. Statistical Methods

In this study, personal information [gender, age group, and health insurance qualification (health insurance or medical aids)] and the number of drug prescriptions, according to the drug standards for each indication, for each patient were analyzed. If one patient was diagnosed with two or more diseases, data for each disease were recorded. If full-labeled, partial-labeled, and contraindicated drugs were prescribed for a single patient, duplicate records were allowed and included in the number of prescriptions in each category. All analyses were performed using SAS software, version 9.4 (SAS Institute Inc., Cary, NC, USA).

## 3. Results

### 3.1. Target Patients and Characteristics

In the pediatric patient dataset provided by HIRA, 19,557 patients had one or more of the following mental health disorders (schizophrenia, bipolar disorder, depressive disorder, anxiety disorder, ADHD, and tic disorder) (Table 2). Mental health disorders were diagnosed more frequently in boys (56.74%) than in girls (43.26%), and mostly among adolescents aged 13–19 years (69.82%) rather than children. A total of 11,966 patients (61.19%) were diagnosed with only one disease, 4955 (25.34%) were diagnosed with two diseases, and 12 (0.06%) were diagnosed with all six diseases.

### 3.2. Patient Characteristics by Disease

When analyzing patients with individual diseases, 8816 patients were diagnosed with major depressive disorder, followed by 8760 with anxiety disorder, and 7263 with ADHD (Table 3). In the case of schizophrenia, bipolar disorder, depressive disorder, and anxiety disorder, adolescent patients accounted for approximately 80% of all patients, but in the case of ADHD and tic disorder, 55% of patients were younger than 12 years of age. In addition, about 80% of those with ADHD and tic disorders and about 60% of those with schizophrenia and bipolar disorder were boys, whereas more girls had depressive and anxiety disorders (Table 3). For anxiety disorder, ADHD, and tic disorder, the rate of diagnosis in preschool-age children was 2–4%, indicating that relatively young children were diagnosed and prescribed medications for these conditions compared to other mental health disorders.

### 3.3. Classification of Prescription Drugs by Indication

All patients diagnosed with psychiatric disorders included in this study were prescribed psychiatric medications. In addition, the analysis confirmed that drugs as full-labeled were used from PI. However, in the case of bipolar disorder, depressive disorder, and anxiety disorder, drugs other than those classified as full-label or partial-label were prescribed for 15–30% of patients (Table 4).

In addition, when drugs classified as partial-label were analyzed, 859 (73.80%), 7438 (84.37%), and 4523 (51.63%) drugs were prescribed for schizophrenia, depressive disorder, and anxiety disorder, respectively. For drugs classified as full-label, 1585 (63.12%), 6506 (89.58%), and 1620 (77.88%) medications were prescribed for bipolar disorder, ADHD, and tic disorder, respectively (Table 4).

In some cases, contraindicated drugs were prescribed for children and adolescents. For major depressive disorder and anxiety disorder, 467 patients (5.30%) and 180 patients (2.05%), respectively, were prescribed contraindicated drugs. In all cases of tic disorder, schizophrenia, and bipolar disorder, drugs that could be classified as contraindicated according to the approval information were prescribed for fewer than 1% of patients (Table 4).

## 4. Discussion

Almost 20,000 pediatric and adolescent patients in the HIRA-PPS dataset had at least one of the following mental health disorders (schizophrenia, bipolar disorder, depressive disorder, anxiety disorder, ADHD, and tic disorder). For the treatment of mental health disorders in children and adolescents, in many cases, cognitive behavioral therapy or counseling therapy is tried first, and if necessary, drug therapy is either added or used instead [11,12,13,14,15,16,17,18]. Therefore, we can estimate that there are actually more children and adolescents with mental health disorders.

In a previous study conducted at a single Korean institution, the male:female ratio of children and adolescents with mental health disorders was 2.3:1, and the most frequently diagnosed disease was ADHD [19]. However, in this study, the number of male children and adolescents diagnosed with a mental health disorder was 56%, even though boy patients diagnosed ADHD were still twice more than girl patients. The previous study also mentioned that the rate of treatment among adolescents increased after 2010 due to social issues such as adolescent suicide compared to preschool-age children. Consistent with this finding, the number of patients diagnosed with depressive disorder or anxiety disorder, which was predominant among the adolescent patients in this study, was high.

The results of this study revealed that drugs that do not have sufficient information provided from clinical trial results or labels are being used to treat mental health disorders in children and adolescents. There could have been ethical difficulties in conducting clinical trials on children and adolescents who are socially disadvantaged, and thus, it would be difficult to specify drug use by age in the indication for use. Additionally, in the case of drugs used for mental health disorders, the approval information for pediatric patients differs for each national regulatory agency. For example, the U.S. FDA (U.S. Food and Drug Administration) has approved risperidone, olanzapine, quetiapine, aripiprazole, and lurasidone for use in children and adolescents over 13 years of age and paliperidone for use in children and adolescents over 12 years of age [20,21,22,23,24,25]. However, in the approval information from the Korean Ministry of Food and Drug Safety (MFDS), clear permission information indicating that these drugs could be used in patients with schizophrenia at these ages was not provided [26,27,28,29,30]. This lack of information was similarly observed for other psychiatric drugs used to treat bipolar disorder and depressive disorder [20,21,22,31,32,33,34]. When looking at this information comprehensively, the MFDS was found to provide less approval information than the U.S. FDA.

For regulatory agencies to obtain drug-use approval for an indication, clinical research or drug-use information supported by valid evidence is needed. In the case of many adults, conducting clinical research is relatively easy compared with that of children, pregnant women, and elderly people. In addition, limiting the approved indications for safe drug use may be necessary. However, for drugs used in actual clinical practice, it is necessary to provide the appropriate approval information. RWE can be used as an alternative to these structural limitations to provide sufficient evidence for the efficacy and safety of drugs used in actual clinical practice. RWE is a method to prepare evidence by collecting and analyzing RWD for information related to the efficacy or safety of drugs used in clinical practice. RWE can be used both before and after drug approval. For example, if it is difficult to conduct a randomized controlled trial for a new approval, such as for a drug for an orphan disease, RWE can be used instead of clinical trial data. Drugs that have already been approved can also be used with RWE for revision or expansion of approval information, pharmacovigilance, etc. [35].

The FDA and European Medicines Agency (EMA) have been using RWD to expand indications and approve new drugs since 1998. Between 1998 and 2018, a total of 17 drugs received new drug approval based on RWD, of which 13 were approved by the EMA, 11 by the U.S. FDA, and two by the PMDA in Japan. Between 2012 and 2018, there were a total of 10 indication expansions based on RWD: seven from the EMA and eight from the U.S. FDA [36,37]. As such, the USA, Europe, and Japan have positively accepted RWD and RWE and are actively using them [21,38,39]. It would be possible to change the labeled information using RWE for pediatric mental health disorders just as it is applied to orphan drugs. In addition, since South Korea has a universal healthcare system and all information (diagnoses, prescribed medication and procedure, or medical expenditure) is collected through HIRA, it could be a sufficient data source as RWD. While such a need is acknowledged, there are few cases of actual permission changes to date in Korea [40]. If RWE is prepared using clinical data, then data demonstrating the efficacy and safety of drugs used in clinical trials should be reflected in the approval. The use of drugs for pediatric mental health disorders particularly may cause adverse reactions different from those of adults [41]. Therefore, using the RWD of pediatric patients for drug evaluation, a pediatric-specific RWE can be established. Further, such a system could be actively used for drugs used to treat mental health disorders in children.

This study had the following limitations. First, because the HIRA data were categorized by age group rather than by the actual patient age, then the ages suggested in the approval information for the drug classifications by indication in this study could not be explicitly applied. Second, full-labeled, partial-labeled, and contraindicated drugs were classified by age for each indication, according to the approval information, and doses according to other patient factors were not taken into account. Third, since this study allowed all overlapping disease codes, the frequencies of full-labeled, partial-labeled, and contraindicated drug prescriptions for each disease differ from the classification of clinical indications. For example, if a 13-year-old patient was diagnosed with schizophrenia and depressive disorder and was prescribed aripiprazole for the treatment of schizophrenia, it would have been classified as full-labeled for schizophrenia but as partial-labeled for depressive disorder. Lastly, galenic preparations which represent a part of the formulation prescribed for psychiatric disorders in children (especially young) were not considered. Nevertheless, this study had several strengths. First, the HIRA data included a sufficient number of children and adolescents undergoing drug treatment for mental health disorders. Therefore, these data could be used objectively to assess drugs used to treat mental health disorders in children and adolescents in Korea. Second, this study was the first to present the current status of drugs prescribed to Korean children and adolescents with psychiatric disorders. Lastly, new drug classification criteria based on clinical guidelines and approval information could clarify how drugs should be used to treat children and adolescents with mental health disorders.

## 5. Conclusions

In conclusion, many children with certain psychiatric conditions are commonly administered drugs that are not explicitly approved for either their disease state or age, including off-label and unlicensed drugs. Expansion of drug information in labels using RWD can be one way to safely use therapeutics for psychiatric disorders in children.

## Figures and Tables

**Table 1:** Classification of antipsychotic medications.

Indication	Generic Name	ATC Code	Full-Label, Years	Partial-Label, Years	CI, Years
Schizophrenia	Aripiprazole	N05AX12	≥13	<13	
	Chlorpromazine	N05AA01	all ages		
	Clozapine	N05AH02		all ages	
	Fluoxetine	N06AB03		all ages	
	Haloperidol	N05AD01	≥13	<13	
	Olanzapine	N05AH03		≥13	<13
	Paliperidone	N05AX13	≥13	<13	
	Perphenazine	N05AB03		≥13	<13
	Quetiapine	N05AH04		all ages	
	Risperidone	N05AX08		all ages	
	Ziprasidone	N05AE04		all ages	
Bipolar disorder	Aripiprazole	N05AX12	≥10	<10	
	Bupropion	N06AX12		all ages	
	Carbamazepine	N03AF01		all ages	
	Escitalopram	N06AB10		all ages	
	Fluoxetine	N06AB03		all ages	
	Lamotrigine	N03AX09		all ages	
	Lithium	N05AN01		all ages	
	Mirtazapine	N06AX11		all ages	
	Olanzapine	N05AH03		≥13	<13
	Paroxetine	N06AB05		all ages	
	Quetiapine	N05AH04		all ages	
	Risperidone	N05AX08		all ages	
	Sertraline	N06AB06		all ages	
	Valproate	N03AG01	all ages		
	Ziprasidone	N05AE04		all ages	
Major depressive disorder	Aripiprazole	N05AX12		all ages	
	Bupropion	N06AX12		all ages	
	Citalopram	N06AB04		all ages	
	Desvenlafaxine	N06AX23			≤19
	Duloxetine	N06AX21			≤19
	Escitalopram	N06AB10		all ages	
	Fluoxetine	N06AB03		all ages	
	Fluvoxamine	N06AB08		all ages	
	Lamotrigine	N03AX09		all ages	
	Lithium	N05AN01		all ages	
	Mirtazapine	N06AX11			≤19
	Paroxetine	N06AB05		all ages	
	Quetiapine	N05AH04		all ages	
	Risperidone	N05AX08		all ages	
	Sertraline	N06AB06		all ages	
	Valproate	N03AG01		all ages	
	Venlafaxine	N06AX16			≤19
Anxiety disorder	Alprazolam	N05BA12		all ages	
	Buspirone	N05BE01		all ages	
	Clonazepam	N03AE01	all ages		
	Diazepam	N05BA01	≥3		<3
	Duloxetine	N06AX21		all ages	
	Fluoxetine	N06AB03		all ages	
	Fluvoxamine	N06AB08		all ages	
	Lorazepam	N05BA06		≥13	<13
	Paroxetine	N06AB05		all ages	
	Sertraline	N06AB06		all ages	
	Zolpidem	N05CF02		all ages	
ADHD	Atomoxetine	N06BA09	≥6		<6
	Bupropion	N06AX12		all ages	
	Clonidine	N02CX02	≥6	<6	
	Imipramine	N06AA02		all ages	
	Methylphenidate	N06BA04	≥6		<6
	Modafinil	N06BA07		all ages	
Tic disorder	Aripiprazole	N05AX12	≥6	<6	
	Baclofen	M03BX01		all ages	
	Clonidine	N02CX02		all ages	
	Haloperidol	N05AD01	≥13	<13	
	Olanzapine	N05AH03		≥13	<13
	Pimozide	N05AG02		all ages	
	Risperidone	N05AX08		all ages	
	Topiramate	N03AX11		≥3	<3
	Ziprasidone	N05AE04		all ages	

CI: contraindication, ADHD: attention deficit-hyperactivity disorder.

**Table 2:** General characteristics of pediatric and adolescent patients with psychiatric disorders.

Characteristics	Patients, *n* (%) (*n* = 19,557)
Gender	
Male	11,097 (56.74)
Female	8460 (43.26)
Age (year)	
Infants (≤2 years)	206 (1.05)
Preschoolers (3–5 years)	368 (1.88)
Childhood (6–12 years)	5329 (27.25)
Adolescents (13–19 years)	13,654 (69.82)
Types of insurance	
Health insurance	17,954 (91.80)
Medical aid	1603 (8.20)
Number of psychiatric disorders diagnosed	
1	11,966 (61.19)
2	4955 (25.34)
3	1940 (9.92)
4	594 (3.04)
5	90 (0.46)
6	12 (0.06)

**Table 3:** Pediatric and adolescent patient characteristics by psychiatric disorder.

Patient Characteristics		Number of Patients (%)					
		SCZ (*n* = 1164)	BPD (*n* = 2511)	MDD (*n* = 8816)	AD (*n* = 8760)	ADHD (*n* = 7263)	Tic Disorder (*n* = 2080)
Gender	Male	698 (59.97)	1480 (58.94)	4308 (48.87)	4053 (46.27)	5624 (77.43)	1668 (80.19)
	Female	466 (40.03)	1031 (41.06)	4508 (51.13)	4707 (53.73)	1639 (22.57)	412 (19.81)
Age group (year)	Infants (≤2)	0 (0)	3 (0.12)	7 (0.08)	197 (2.25)	1 (0.01)	1 (0.05)
	Preschoolers (3–5)	10 (0.86)	19 (0.76)	78 (0.88)	142 (1.62)	181 (2.49)	39 (1.88)
	Childhood (6–12)	208 (17.87)	530 (21.11)	1373 (15.57)	1104 (12.60)	3950 (54.39)	1163 (55.91)
	Adolescents (13–19)	946 (81.27)	1959 (78.02)	7358 (83.46)	7317 (83.53)	3131 (43.11)	877 (42.16)
Type of insurance	Health insurance	1003 (86.17)	2212 (88.09)	8043 (91.23)	8135 (92.87)	6534 (89.96)	1946 (93.56)
	Medical aid	161 (13.83)	299 (11.91)	773 (8.77)	625 (7.13)	729 (10.04)	134 (6.44)

SCZ: schizophrenia, BPD: bipolar disorder, MDD: major depressive disorder, AD: anxiety disorder ADHD: attention deficit-hyperactivity disorder.

**Table 4:** Frequency of prescribed medication according to regulatory information by psychiatric disorder.

Category of Medication Use	Number of Patients (%)					
	SCZ (*n* = 1164)	BPD (*n* = 2511)	MDD (*n* = 8816)	AD (*n* = 8760)	ADHD (*n* = 7263)	Tic Disorder (*n* = 2080)
Full-label	628 (53.95)	1585 (63.12)	0 (0.00)	2943 (33.60)	6506 (89.58)	1620 (77.88)
Partial-label	859 (73.80)	180 (7.17)	7438 (84.37)	4523 (51.63)	349 (4.81)	645 (31.01)
Contraindication	2 (0.17)	9 (0.36)	467 (5.30)	180 (2.05)	131 (1.80)	1 (0.05)

SCZ: schizophrenia, BPD: bipolar disorder, MDD: major depressive disorder, AD: anxiety disorder ADHD: attention deficit-hyperactivity disorder.

## Data Availability

The data presented in this study are available upon request from the corresponding author.
